# Limitations and solutions of low cost virtual reality mirror therapy for post-stroke patients

**DOI:** 10.1038/s41598-023-40546-2

**Published:** 2023-09-07

**Authors:** David Quintana, Antonio Rodríguez, Imma Boada

**Affiliations:** Graphics and Imaging Laboratory, Institut Informàtica i Aplicacions, Universitat de Girona, Campus de Montilivi, 17003 Girona, Catalunya Spain

**Keywords:** Health services, Quality of life, Health care

## Abstract

Mirror therapy is applied to reduce phantom pain and as a rehabilitation technique in post-stroke patients. Using Virtual Reality and head-mounted displays this therapy can be performed in virtual scenarios. However, for its efficient use in clinical settings, some hardware limitations need to be solved. A new system to perform mirror therapy in virtual scenarios for post-stroke patients is proposed. The system requires the patient a standalone virtual reality headset with hand-tracking features and for the rehabilitator an external computer or tablet device. The system provides functionalities for the rehabilitator to prepare and follow-up rehabilitation sessions and a virtual scenario for the patient to perform rehabilitation. The system has been tested on a real scenario with the support of three experienced rehabilitators and considering ten post-stroke patients in individual sessions focused on upper limb motor rehabilitation. The development team observed all the sessions and took note of detected errors regarding technological aspects. Solutions to solve detected problems will be proposed and evaluated in terms of feasibility, performance cost, additional system cost, number of solved issues, new limitations, or advantages for the patient. Three types of errors were detected and solved. The first error is related to the position of the hands relative to the head-mounted display. To solve it the exercise area can be limited to avoid objectives that require turning the head too far. The second error is related to the interaction between the hands and the virtual objects. It can be solved making the main hand non-interactive. The last type of error is due to patient limitations and can be mitigated by having a virtual hand play out an example motion to bring the patient’s attention back to the exercise. Other solutions have been evaluated positively and can be used in addition or instead of the selected ones. For mirror therapy based on virtual reality to be efficient in post-stroke rehabilitation the current head-mounted display-based solutions need to be complemented with specific strategies that avoid or mitigate the limitations of the technology and the patient. Solutions that help with the most common issues have been proposed.

## Introduction

Rehabilitation is defined as “a set of interventions designed to optimize functioning and reduce disability in individuals with health conditions in interaction with their environment”^1^. The improvements in technology and the affordability of provided solutions have generated an increasing interest in the use of Virtual Reality (VR) and gaming technology for rehabilitation purposes^2–6^. Conventional methods are being substituted by virtual gamified tasks aimed to maintain patient interest and motivation to enhance recovery^7,8^. In this way, using off-the-shelf solutions or specifically designed systems new rehabilitation environments with patients and rehabilitators as the main end-users are created^9,10^. In this paper, we will focus our interest on post-stroke rehabilitation that uses VR head-mounted displays (HMD) as the main component of the rehabilitation scenario. HMD equipped with multiple built-in cameras can track user movements allowing them to naturally interact with the objects of the virtual environment^11^. As a result, patients are immersed in safe, controlled, and engaging virtual worlds where rehabilitation tasks can be carried out^12^.

VR interventions in post-stoke rehabilitation sessions use to be in the form of gamified exercises (grouped in sessions) that take place in a virtual scenario^13^. The patient using an input device will interact with the scenario to achieve a predefined goal while receiving some feedback as output via visual and audio channels or vibration depending on the device. The patient interaction will require some specific actions whose repetition will contribute to the recovery. In this regard, actions such as press, touch, grab, move/drag, and place made by themselves or combined, and considering or not an object are the common ones. In addition, these VR systems will require different devices and specific software components to support rehabilitators in the creation of rehabilitation exercises, and patient follow-up. Particularly, three main components can be considered: (i) a Session Preparation component with functionalities to create and prepare rehabilitation exercises and sessions. Depending on software capabilities the range of functionalities of this module can vary from simple access to predefined exercises to advanced software tools that allow the creation of virtual scenarios, configuration of interaction parameters and feedback messages, etc. Authoring tools or specialized editors are of special interest for the advantages that provide to end-users^14–17^. Regarding required devices, this module generally runs on a computer, tablet, or smartphone; (ii) A Session follow-up or Monitoring module to control patients’ performance in the virtual scenario and return information to the rehabilitator for proper follow-up^18^. As in the previous case, depending on the used software and hardware, provided functionalities can range from a simple display of patient execution to the possibility for the rehabilitator to interact with the scene. By default, in the case of HMD-based rehabilitation, the software device can be used for monitoring purposes. However, there are also more advanced solutions able to support telerehabilitation^19,20^ and other features; (iii) a Virtual scenario where the patient will perform rehabilitation. In HMD-based rehabilitation, this device maintains the scenario and transforms patients’ movements into virtual actions^21,22^. In post-stroke upper limb rehabilitation, hand tracking is an essential part to be considered, being necessary for the patient’s hands rendering and tracking in real-time during exercise performance^23,24^. The integration of mirror techniques in these environments is of special interest due to the benefits that can bring to post-stroke patients^25,26^.

Mirror therapy (MT) is a well-known therapy initially used for amputees to reduce phantom limb pain^27^. Placing a mirror in the middle of the body’s patient, such that the affected part is hidden, the movements of the non-affected side appear as a reflection of the affected one. The created illusion that the amputated member does the same movement as the non-amputated one leads the patient to experience tingling or paresthesia, i.e., a sensation in the affected limb, that reduces pain^28^. The benefits of this therapy have been extended not only to reduce phantom pain but also to improve the movement of the affected limbs in post-stroke patients^29–31^. The created illusion with the mirror stimulates damaged nerve connections to make reconnections that benefit patient recovery^32^. Studies have also demonstrated the positive effects of MT on sensory and perception deficits, among others^31^.

Focusing on MT performance, the key element for its execution is the mirror which depending on the size determines the movements and activities that can be carried out. In addition to the classical approaches, there are also more advanced solutions based on virtual reality (VR)^33^.

To recreate MT, in rehabilitation sessions, patients are instructed to complete different activities using their unaffected extremity while maintaining the affected one in a relaxed state (or magnifying movements when possible^34^). The system captures movements of the healthy hand (or limb) and projects them to the affected extremity in the virtual scenario. This approach supports different configuration options such that the visualization of both hands or only one, adapting the position of the mirror, etc. Moreover, the therapist according to the patient’s status and using the software that accompanies the VR framework can select the option that better fits the patient (unilateral/bilateral interaction, mirroring/not-mirroring, etc.).

Different proposals for MT based on VR (VR-MT) have been proposed. The first ones required HMD with hand controllers^25^ while the more recent substitute these controllers with Leap Motion creating more comfortable scenarios for the patients^33^. There are also systems that combine HMD with external sensors that better collect patients’ movements^26,35^. Independently of used technologies, VR-MT techniques have generally been evaluated with the patient as the main focus of interest^36^ while technical aspects have not been taken into account. However, from a practical point of view, these aspects need to be considered to ensure the correct performance. The aim of this paper is to present a VR-MT system and the main technical issues detected during its evaluation as well as the proposed solutions. The evaluation has been done in real post-stroke rehabilitation sessions considering ten patients in individual rehabilitation sessions, with the support of three experimented rehabilitators and being observed by the development team.

## Material and methods

### Proposed VR-MT system

#### System requirements

The requirements have been defined in collaboration with the medical team involved in the project. To define the requirements, two user profiles have been considered: the rehabilitation team which is composed of doctors, nurses, physiotherapists, etc., and is responsible for the preparation of rehabilitation sessions and their follow-up, and the post-stroke patients who have to perform the exercises defined by the experts in the context of a rehabilitation session.

Since rehabilitation exercises are the key to the proposed system, we will start presenting their requirements. Particularly, *rehabilitation exercises* have to:

- Take place in a virtual scenario that can be explored using VR with a consumer-grade headset;
- Be presented as games to motivate patients to practice with challenges to overcome and rewards to receive according to performance;
- Be interactable with hand movements as the main interaction strategy to achieve exercise goals;
- Support mirror therapy with different options to place the mirror;
- Support different levels of difficulty to fit different patient profiles;
- Provide different feedback strategies to fit patient needs.

Regarding rehabilitators, the proposed system has to allow the *rehabilitation team* to:

- Set exercise parameters regarding feedback, visualization mode, and the text information that will be provided to the patient;
- Configure the interaction required to achieve the exercise goals focusing on hand actions;
- Configure the mirror position and whether it is fixed or follows the patient’s head or the patient’s hands;
- Configure the skin and nails of virtual hands to improve realism;
- Create rehabilitation sessions with different exercises;
- Configure the session length by giving the therapist the choice of how long (seconds/minutes) the play session will run for;
- Configure the target difficulty, such as the time between subsequent obstacles, or the physical distance between obstacles;
- Configure the hands, including visibility of each hand, if they can interact with the virtual world, and the magnification of the motions^37^;
- See the state of the rehabilitation session, interact with the objects on behalf of the patient, and change some of the configuration values during the session;
- Obtain information on patient progress and performance;
- Test exercises to see how the patient will see them.

Finally, focusing on *the patients* the system has to allow them to:

- Perform rehabilitation exercises in a game mode;
- Receive feedback on the actions that have to be carried out and on their performance;
- Adapt the level of challenge to the patient’s performance.

#### System architecture

**Figure 1:**
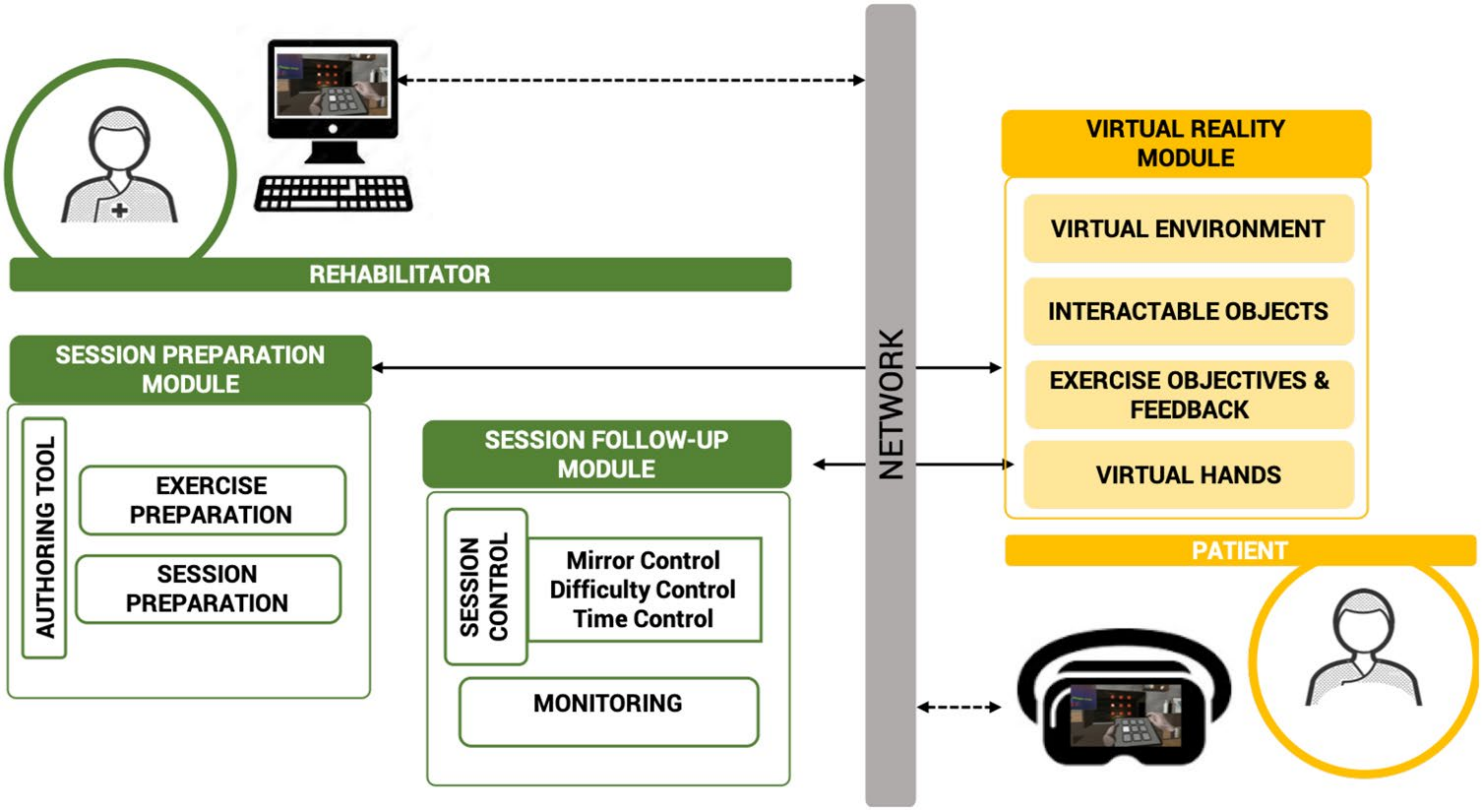
The main modules of the proposed VR-MT system to satisfy the rehabilitator and patient’s requirements.

Taking into account the defined requirements, the system presented in Fig. 1 and described below is proposed. Since hemiparesis of the upper extremity is the most common disability in post-stroke patients^38^, the first version of this system has been designed for upper limb motor rehabilitation.

For its proper performance, the system requires the patient a standalone VR headset with hand-tracking features. In our case, for its low cost, availability, ease of development, and the use of camera-based hand tracking, the Oculus Quest device has been selected. However, other similar devices that exist in the market can be used with similar capabilities and limitations. For the rehabilitator, the system requires an external computer or tablet device. Note from Fig. 1 that these devices are connected via the local network or the internet.

#### Session preparation module

**Figure 2:**
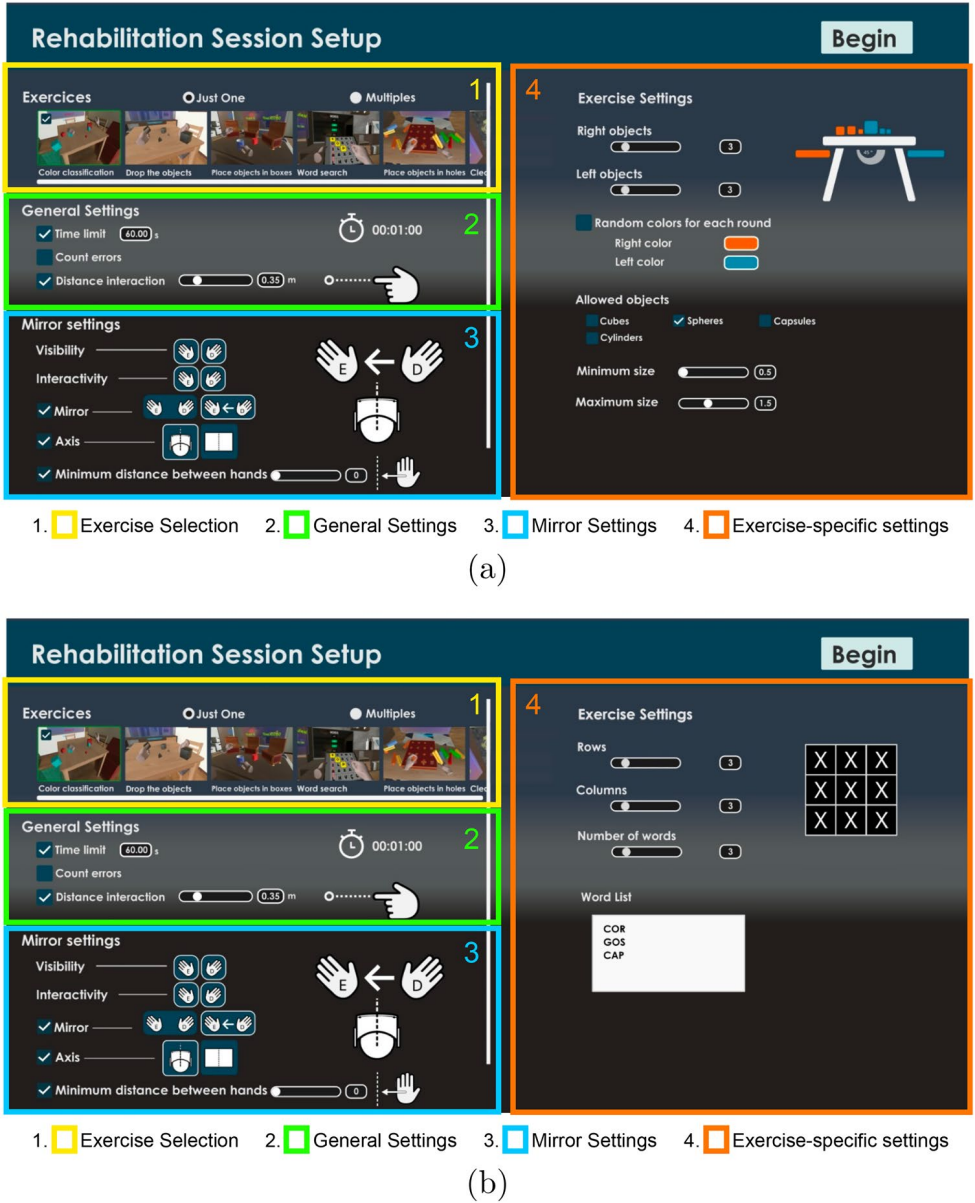
Menus of the authoring tool to prepare rehabilitation sessions corresponding to an activity where: (**a**) the defined number of objects have to be classified by color and (**b**) the entered words by the rehabilitator will appear as images that will have to be identified by the patient in the word panel.

The Session Preparation module provides functionalities for the rehabilitator to prepare rehabilitation exercises and organize sessions. The tool is composed of different menus (see Fig. 2) that provide, for instance, access to the repository of exercises (represented by an image that shows the scenario and the action to be carried out). The rehabilitator selects the exercises of the session and for each exercise, the parameters to be set are: (1) the hand’s visibility which can be (i) unilateral or (ii) bilateral; and (2) the position of the mirror which can be: (i) fixed in the scene or (ii) relative to the patient’s head. It is also possible to set a distance restriction to avoid the patient to traverse the mirror plane from one side to the other; (3) The hand’s ability to interact with objects in the exercise, such that a non-interactive hand will behave more like a ghost hand, if visible, and pass through objects. In addition, as shown on the right side of the menu, the parameters of the selected exercise can also be defined. There are also menus to define parameters such as the feedback text, the time assigned to the patient to solve the activity, or the reward system according to the errors. It is also possible to determine the color of hands and nails.

#### Session follow-up module

The Session Follow-up module allows to control patient performance and, in some cases, modify configuration parameters if required. To provide these functionalities, in the simplest case, the official software of the HMD, such as the Meta Quest app^39^, can be used from a standard mobile device to cast the image from the HMD and display it on the mobile device. There is also more advanced software that can be used which allows interacting with the virtual world and adjusting difficulty parameters, enabling telerehabilitation^40^. The details and limitations of this module, in its basic or advanced forms, are out of the scope of this article and not considered here.

#### Virtual reality module

The Virtual Reality Module has been designed with a focus on the patient. This provides the virtual scenario which has been created following the rehabilitator’s indications. It is assembled from a number of elements following the hierarchical structure of the Unity game engine^41^. The virtual scenario is hosted by a Scene, in which the chosen environment (e.g., the interior of a house) is placed. In this environment, decorative objects can be placed, and then moved and rotated as needed. An exercise surface is chosen, such as the top of a table or a section of wall, in which the exercise will be located. Once the exercise starts, the chosen exercise will then place the objects on that exercise surface based on the configuration of the exercise. Objects can optionally be interactive, and have physics behaviors enabled. Objects can have objectives assigned, such as the object being placed inside a target area, and rewards can be assigned for performing these objectives, such as granting points, or feedback.

### VR-MT system evaluation

The system evaluation has been done with the support of three experimented rehabilitators that participated in the project. Using the system editors, the rehabilitators created a rehabilitation session composed of seven exercises. These exercises require basic hand and arm movements to be solved that reproduce the movements patients do in their no virtual rehabilitation sessions^42–44^.

#### Rehabilitation session exercises

**Figure 3:**
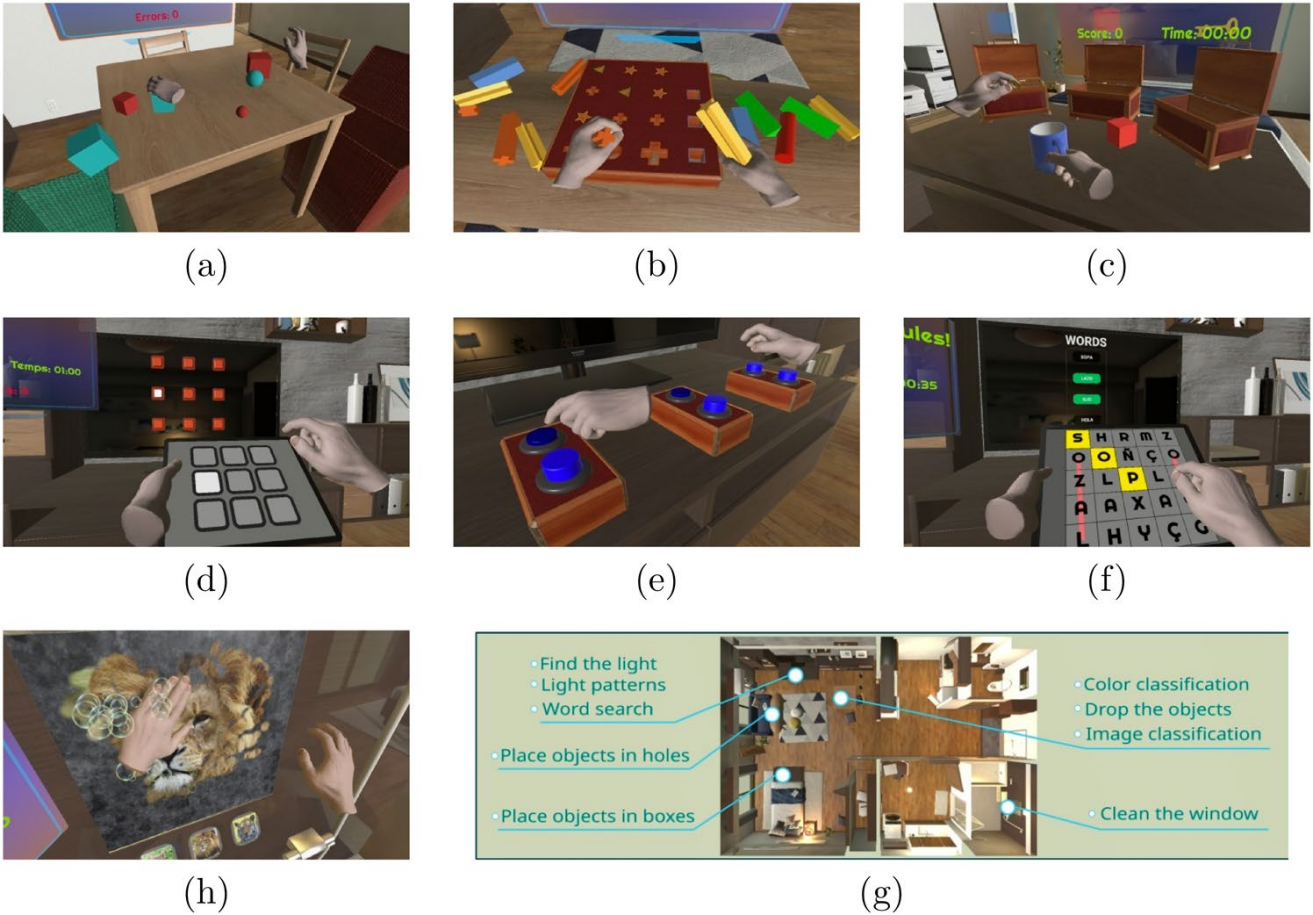
The exercises that compose the rehabilitation sessions: (**a**) Color classification, (**b**) Place objects in holes, (**c**) Place objects in boxes; (**d**) Light patterns, (**e**) Find the light, (**f**) Word search, and (**g**) Clean the window; and (**h**) a global view of the virtual scenario where the exercises take place.

The seven exercises that compose the rehabilitation session are illustrated in Fig. 3 and described below.

- Classify objects by color. This exercise consists of a number of objects of two different colors placed on a table, and two open boxes on either side of the table matching each of the colors. The goal consists on using the hands to *move/drag* the objects so they fall into the correct box (see Fig. 3a).
- Place objects in shaped holes. This exercise consists of a number of objects matching one of the shaped holes in a box that is placed in the center of the table. The goal is to *grab* and *place* each object in the right hole. This exercise allows using a grab gesture to pick the object, for ease of manipulation (see Fig. 3b).
- Place objects in boxes. This exercise consists of a number of closed boxes with a hinging lid, and a corresponding number of objects, all on a table. Above each box is a ghost image of one of the objects. The goal is to *grab *and open the lid and *place* the correct object inside the box (see Fig. 3c).
- Reproduce light patterns. This exercise consists of a set of on/off squares projected in a plane and another plane with the same number of squares but all of them off. The goal is to *press* squares to reproduce the projected pattern (see Fig. 3d).
- Find the light. This exercise consists of a number of light-up buttons scattered across a table. One button lights up at random and the goal is to find and *press* this button, after which another button will light up (see Fig. 3e).
- Word search. This exercise consists of a holographic letter grid and a word display. A number of words are displayed above the letter grid, and the goal is to use *pressing* gestures to activate adjacent letters in a row, column, or diagonal, like in the pen and paper game (see Fig. 3f).
- Clean the window. A mirror, window, or picture frame is shown with some dirt or condensation covering it up. The hand *touches *the window and *moves* across it, in order to clean the window and reveal an image. This image contains the clues needed to *press* the right answer to a question (see Fig. 3g).

### Testing scenario

Created exercises have been used in rehabilitation sessions for acute stroke patients with moderate to severe upper limb impairment. Each exercise required approximately five minutes to be solved. The rehabilitation sessions started at a set time when the patients arrived at the rehabilitation room. The rehabilitator prepared a Meta Quest 2^45^ device by turning it on and running the software. Then, an explanation was given to the patient indicating the goal of each exercise. In addition, exercises provide textual descriptions of the goals. After the explanation, the patients were equipped with the HMD and rehabilitators monitored the patient via a tablet device running the Meta Quest software^39^, which allows casting a video feed from the VR Headset into the mobile device.

Ten patients in individual rehabilitation sessions were considered for the tests. The same session composed of seven rehabilitation exercises was prepared for all the patients. To support the patient one of the three experimented rehabilitators was always present in the session. All the exercises were configured with the same level of difficulty. The development team was present in all the sessions as observers with no interaction with the patient. During patient performance, the team took note of detected errors regarding technological aspects.

#### Ethical approval

The project and the current study were approved by the Ethical Research Committee of the CEIC GIRONA clinic, with CEIM code 20201.051 in accordance with the current legislation (Spain law 14/2007 for the biomedical research, Royal Decree 1090/2015 of the 4th of December, and European Regulation 536/2014 of the 16th of April, through which clinical assays with medicine are regulated). Informed consent was obtained from all participants included in the study.

## Results and discussion

This section has been structured in three parts. The first one summarizes the observations of the development team focusing on the errors that have been detected in the rehabilitation sessions and their possible causes. The second part focuses on the solutions that can be applied to tackle detected problems. The last part presents some final remarks.

### Detected issues during rehabilitation sessions

The development team acting as observers of the rehabilitation sessions collected the issues that were detected during the patient’s performance. These issues were grouped into three main categories labeled C-I, C-II, and C-III, and representing issues related to the position of the hands relative to the HMD; issues related to the interaction between the hands and the virtual objects; and issues caused by limitations in the patient, respectively. As shown in Table 1, where the relationship between patient, sessions, and issues is collected, except in the *Clean the Window* exercise, these issues appear in almost all the exercises and for almost all the patients.

**Table 1:** For each rehabilitation exercise, the number indicates how many patients have made the error indicated in the column name.

Category	C-I	C-II	C-III
Source of issues	Hands position relative to HMD	Hands-objects interaction	Patient limitations
Color classification	8	9	9
Objects in holes	4	8	6
Objects in boxes	2	8	7
Light patterns	0	6	4
Find the light	4	9	3
Word search	1	8	1
Clean the window	0	0	8

**Figure 4:**
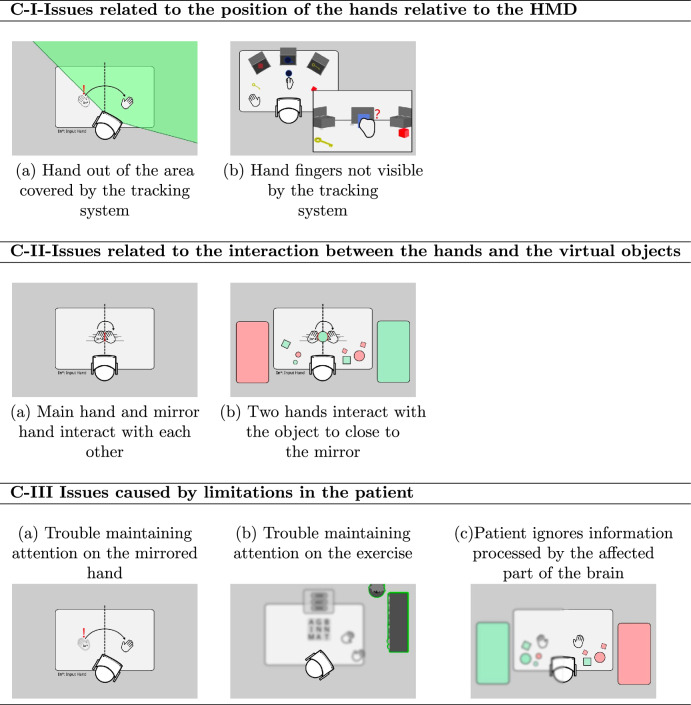
The three categories of issues detected in rehabilitation sessions.

To better present C-I, C-II, and C-III categories, in Fig. 4, the main issues for each category are illustrated. From top to bottom, the C-I category of Fig. 4, issues related to the position of the hands relative to the HMD, considers two issues that have been detected when: (a) the patient turns the head to look at an object that is far away from the virtual mirror, it is possible for the main hand to leave the view range, at which point the hand tracking system cannot continue providing valid information; and (b) the hand becomes turned in a way that hides the fingers and the hand tracking system cannot continue to provide valid information. As an example of these situations consider the *Classify objects by color* exercise, where a number of objects are scattered on a table, during the testing sessions, the patients would follow the mirrored hand with their sight and turn their heads to watch the hand as it carried the objects. In some cases, this resulted in the healthy hand going out of range of the head tracking cameras, at which point the system failed to continue the tracking, and the hand disappeared from the virtual world. This confused some patients, who did not realize they had to turn their heads back to a more central position and move their hands roughly in front of them for the system to detect the hand again. From Table 1 it can be seen that this error does not appear in *Light patterns* or *Clean the window*. A possible explanation is the fact that in these exercises the patient has to concentrate on a central point of the scene while in the other exercises, a wider range of interaction is required.

The C-II category of Fig. 4, issues related to the interaction between the hands and the virtual objects, considers two issues that arise when the mirror and the main hand are both visible and interactive. Detected issues appear: (a) when the main hand is close to the virtual mirror the main hand and mirror hand interact with each other preventing the hands from continuing to move and follow the patient’s movements; (b) similarly if an object is pushed or held by one hand, this object will interact with the other hand once the object gets close to the virtual mirror, preventing the object from being moved to the other side of the mirror. As an example of these situations consider the place *Objects in boxes* exercise, where a number of objects have to be placed in their corresponding boxes. During the test sessions, the patients sometimes would need to grab an object that was randomly located within the table on either side of the mirror and bring it to a box that was on the other side of the mirror. This caused problems because the mirrored hand and the healthy hand would collide with each other at the mirror plane. From Table 1 it can be seen that this error appears in almost all exercises except the *Clean the window*.

Finally, the C-III category of Fig. 4, issues caused by limitations in the patient, considers the issues that arise when: (a) the patient has trouble maintaining attention on the mirrored hand, (b) the patient has trouble maintaining attention on the exercise, and (c) the patient ignores information processed by the affected part of the brain. As an example of the issue (a) consider the *Place objects in shaped holes* exercise. Some of the patients would reach for objects using their healthy hands, instead of the mirrored one. As an example of the issue (b) consider the *Word search* exercise. One of the patients did not maintain attention on the letter board, and instead looked at their surroundings and tried to interact with decorative objects. As an example of the issue (c) consider the *Find the light* exercise. One of the patients did not recognize when the light on the far left was turned on and was convinced none were lit up, and needed to be told to try looking in that direction, requiring more attention from the rehabilitator.

### Possible solutions to detected issues

The different solutions that can be applied to solve detected problems are described in this section. For the sake of clarity, they are grouped following the error categories C-I, C-II, and C-III illustrated in Fig. 4. In addition, to the solution description, we will evaluate each solution considering the measures presented in Table 2. The first group of these measures considers the implementation cost of the proposed solution in terms of *feasibility*, i.e., the research and development that would need to be done, and *performance*, i.e., the computation cost on performance, and the *system cost*, i.e., the hardware required to support the solution. The second group of measures consider the *issues of each category that have been solved* and the *new limitations that are introduced* with the proposed solution. Finally, the impact on the patient measured as *patient experience* is also considered. For each measure, the possible values that can take are also presented. These values have been defined considering the two extreme cases and one or two as intermediate ones.

**Table 2:** Description and possible values of the measures that have been considered to evaluate possible solutions to the errors detected during rehabilitation sessions.

Measure	Description	Possible values
Feasibility	Complexity of implementation using current low-cost HMD technology and accessories (Higher is better)	*Low* technology not available *Medium* technology available but costly *High* technology readily available
Performance cost	Computational cost on performance (Lower is better)	*None* no performance penalty *Low* small performance cost *Moderate* intermediate performance cost *High* require external calculations
System cost	Additional hardware cost on performance (Lower is better)	*None* no additional hardware *Low* cost $\le 50$ euro *Moderate* 50 euro < cost $\le 100$ euro *High* 100 euro < cost $\le 500$ euro *Very High* cost $> 500$ euro)
Issues solved	The solution fixes all the issues (All is better)	*Some (listed)* major issues unsolved or partially solved *All* issues in the category are solved
New limitations	New limitations are introduced (None is better)	*None* *Minor* limitations do not affect rehabilitation *Major* limitations have a substantial impact
Patient experience	How the solution affects the patient (Positive is better)	*Negative* exercises are harder to perform *Positive* solutions would aid the patient *Mixed* solutions have pros and cons *Neutral* solutions do not affect

#### Solutions to hands position relative to the HMD issues

This category of issues is related to the position of the hands relative to the HMD (see Fig. 4 C-I(a) and C-I(b)). To solve them six possible strategies can be applied:

(i) *Adding an external camera*. This solution would provide a second viewpoint on which to perform hand tracking. This would allow tracking the hands even when they are not visible to the HMD, but it would come with its own limitations due to the fixed position of the external camera^46^. The additional processing needed would have a high computational cost so the external camera would need to be processed by an external computer, and the hand-tracking information communicated via networking.
(ii) *Positional feedback*. This solution would add a feedback system that notifies the patient when their head turns too far. This would not fix the issue *per se*, but it would allow the patient to know that they are out of range and should turn their head back to the mirror.
(iii) *Limiting the exercise area*. This solution would avoid objects from being placed too far away from the virtual mirror. The main drawback of this is that it greatly reduces the available motion range.
(iv) *Alternative hand tracking algorithms*. This solution would use a third-party library for hand tracking algorithms^47^, which may provide more stable or robust results and may be able to guess the position of the fingers/hand even if they are not directly visible. However, this solution is not valid for the case of the Meta Quest headset, because the HMD cameras are reserved for the Meta Quest SDK libraries and are not directly available as video sources to application developers. Although it would be possible to use an external camera for this, the benefits would be low, compared to placing the external camera away from the HMD.
(v) *Using VR controllers*. These are the controller devices that are provided along with the commercial HMDs. The solution allows tracking the hands in a way that does not rely on computer vision, but it greatly limits the movements of the patient and requires that the patient is able to grip the controller. Because of this, we consider it a cumbersome option and negative for the patient’s rehabilitation experience, despite having no additional cost.
(vi) *Using VR haptic gloves*. These gloves contain hardware that tracks the position of the hand in a similar way to the VR controllers, but also have individual position trackers in the glove fingers, and can report this information to the HMD^48,49^. In addition, haptic gloves can apply force against the attempted motion of the hand, simulating the size, shape, and material properties of an object. These gloves are highly expensive, ranging in multiple thousands of Euro, making them a very high-cost solution. In addition, the gloves may be hard to put on or remove for patients with limited hand mobility, making them a negative experience overall^50^.

**Table 3:**
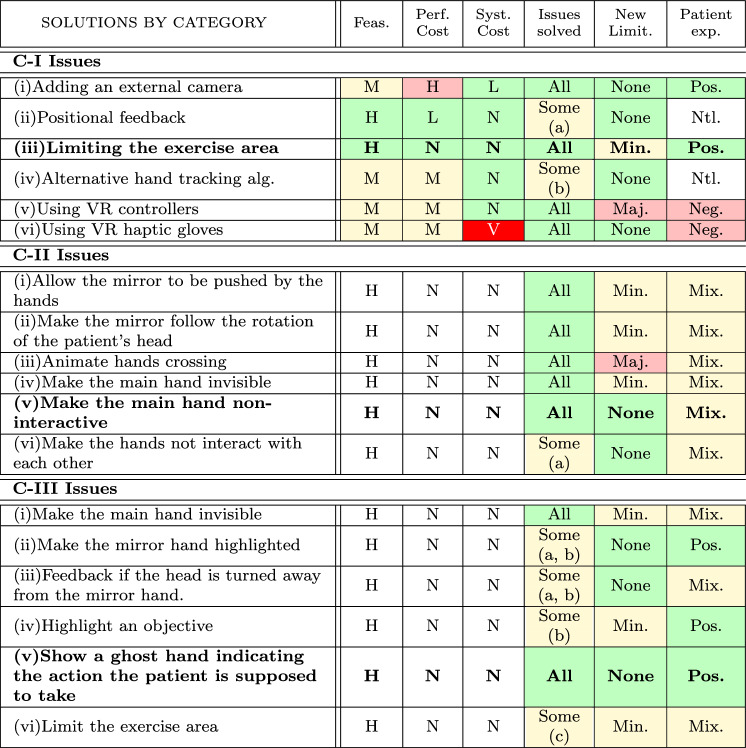
The evaluation of solutions for each category of the errors using measures of Table 2 (Feasibility(Low, Medium, High), Performance Cost and System Cost (None, Low, Moderate, High, Very High, Issues solved (All, Some (lists the issues as described in Section 3.1)), New Limitations (None, Minor, Major), and Patient experience (Neutral, Negative, Mixed, Positive)

The color of the cell indicates if the value contributes positively or negatively to our evaluation (white - does not contribute, green - positive, yellow - mild negative, red - strong negative, intense red - very strong negative)

### Solutions to position of the hands relative to the virtual world issues

This category of issues is related to the position of the hands relative to the virtual world (see Fig. 4 C-II(a) and C-II(b)). To solve them six possible strategies can be applied:

(i) *Allow the mirror to be pushed by the hands*. This means that if a hand gets too close to the mirror, the mirror moves away from the hand. When two hands are visible, it is not clear if the mirror should be controlled by the main hand or the mirror hand. In addition, it is possible that moving the mirror will cause confusion in the patient.
(ii) *Make the mirror follow the rotation of the patient’s head*. This solution makes the mirror move automatically in response to the patient’s head turning side to side, to align with the direction of the head. Because the position of the hands relative to the mirror has changed, this causes the mirror hand to change position without the patient making any movement of the hands. This effect can be partially mitigated by applying smoothing and movement thresholds to avoid constant movements and very rapid movements, but even with this it is possible that this solution could cause confusion and disorientation in the patient, or a loss of sense of the mirror hand being their own.
(iii) *Animate hands crossing*. This solution would add an animation of the hands crossing over and moving to the opposite side of the virtual mirror. This animation would start when the hands are moving in the direction of the mirror and attempting to cross it. This means that when the target is very close to the mirror, the animation will place and prevent grabbing or interacting.
(iv) *Make the main hand invisible*. This would also disable the interaction of the invisible hand with objects in the scene. This solution fixes the issue of hands interacting with each other since there is only one hand. However, since the patient will look at the mirror hand, it is more likely that the main hand will be out of range of the hand-tracking cameras.
(v) *Make the main hand non-interactive*. This would only disable the interaction of the main hand with objects in the scene and would display the hand in a special way, such as translucent, to indicate that it is not meant to be used.
(vi) *Make the hands not interact with each other*. In addition, a potential solution has been considered where the virtual hands do not interact with each other, and only interact with the virtual objects. This would allow the hands to pass through each other, but it would not solve the issue of an object getting trapped between the hands.

#### Solutions to issues related to the limitations of the patient

This category of issues is related to the limitations of the patient (see Fig. 4 C-III(a), C-III(b), and C-III(c)). The following strategies can be used as potential solutions:

(i) *Make the main hand invisible*. This solution is the same as presented in category (II) solution (iv) and shows similar limitations. With regards to this issue, having only one hand should make it easier for the patient to maintain focus on the mirror hand.
(ii) *Make the mirror hand highlighted*. This solution makes the mirror hand highlighted in order to draw attention to it. Blinking or a heartbeat effect (where the light becomes stronger and weaker in a smooth way, instead of blinking on and off) can be applied to increase the effect further.
(iii) *Feedback if the head is turned away from the mirror hand*. This solution detects when the head is turned away from the mirror hand, and provides some form of feedback. This feedback can be presented in multiple ways, such as some kind of highlight/blinking on the mirror hand, an animation of a ghost hand pointing in the right direction, or an arrow.
(iv) *Highlight an objective*. This solution would make the next objective (or a randomly selected objective) light up or blink.
(v) *Show a ghost hand indicating the action the patient is supposed to take*. This solution would use the same system used for exercise demonstrations and tutorials and would perform an example movement as a way to draw attention to the objectives.
(vi) *Limit the exercise area*. This solution would use a reduced exercise area, reducing distracting elements outside this area, so that the patient’s attention can be better focused on the exercise^13^.

All solutions except the last one are feedback strategies, and they will be evaluated primarily against limiting the exercise area.

### Final remarks

To conclude this section in Table 3 the evaluation of each solution using the proposed measures is summarized. For the sake of simplicity, we will emphasize on the solution considered as the best one although references to other options will be also given since more than one solution is possible. From this table it can be seen that, for C-I issues, solution (i), using an external camera, would have a considerable computational cost, which is beyond what is achievable by a standalone headset that is already loaded by the rehabilitation exercises; solution (ii), does not fix all the issues since the hands still lose track; solution (iii), limiting the exercise area, has no cost, and only requires being mindful when creating the exercise, however due to the limited area, there is less range of movement being exercised; solution (iv) is only viable with the use of an external camera, with similar limitations to solution (i); solutions (v), using the VR controllers, and (vi), using haptic gloves, are considered a negative experience for the patient, and solution (vi) has a very high cost on top of that. Based on the evaluation results, solution (iii) is the one that provides the most advantage with the least drawbacks, but solutions (ii) and (iv) are good alternatives. Since the feasibility parameter applies mostly to R &D, if the added cost of an external tracking camera and a small computer to process the tracking data is not a concern, then solution (i) would take the top spot.

For C-II issues, solution (i), push the mirror with the hands, has a limitation where if the mirror is set to be pushed with one hand, the other hand would move by itself when the mirror moves; solution (ii), making the mirror move with the patient’s head, has the same issue but worsened by the fact that the mirror moves any time the patient head has changed direction, which may be especially problematic if they are trying to look at the mirror hand and it keeps moving away; solution (iii), animate the hands crossing over each other, has been evaluated as mixed for the patient experience, but with this is because it has one major limitation, where objects placed close to the center of the screen may be hard to interact with due to the animation, but in cases where this limitation could be avoided, the experience would be positive; solution (iv) makes the main hand invisible, showing only the mirrored hand. this has been considered a limitation since the patient isn’t seeing the hand they are actually moving; solution (v), making the main hand non-interactive, has been proposed as a compromise between fully interactive and fully invisible, which allows the patient to still see their hand, but be incentivized to use the mirror; and solution (vi), make the hands not interact with each other would fix the issue of the hands getting stuck at the mirror, but only with empty hands, if an object is being carried by one of the hands, then the other hand will still interact with the object. Based on the evaluation results, solution (v) has been considered the most promising, but solutions (i) or (ii) could be applied alongside (v), and solutions (iv) or (vi) could be used instead of (v) if some other issue prevented the use of (v).

For C-III issues, solutions (i) to (v) are all feedback strategies, while solution (vi) limits the exercise area. In solution (i), make the main hand invisible, the same limitations to C-I(iii) apply; solution (ii), make the mirror hand highlighted, does not introduce any limitations and gives a positive experience, but only partially solves the issues; solution (iii), feedback when the head is turned away from the mirror hand, does not introduce new limitations, but could annoy the patient; solution (iv), highlight an objective, only solves some of the issues; solution (v), showing a ghost hand giving an example of interaction, can be used to draw attention to the relevant location, and does not introduce new limitations; solution (vi), limit the exercise area, could be useful for extreme cases, but the reduced area limits the advantages of VR rehabilitation. Based on the evaluation results, a feedback strategy is preferred over limiting the exercise area, and out of the evaluated options, solution (v) has been considered the best. All the other solutions are also considered acceptable and can be used in addition to, or instead of (v).

To end this section, we want to highlight that multiple solutions can be combined, such as the use of an external camera (C-I(i)) along with limiting the exercise area (C-I(iii) & C-III(vi)). We also want to mention that detected errors are not specific to our solution but general to the application of hand-tracking techniques in the context of virtual reality applications^51^.

## Conclusions and future work

In this paper, a new system to support Virtual Reality based Mirror Therapy has been presented and technically evaluated in a real scenario with post-stroke patients. From this evaluation, a number of issues have been detected and grouped into three categories related to (i) the position of the hands with respect to HMD; (ii) the interaction between the hands and the virtual objects; and (iii) the limitations in the patient. For each category, potential solutions have been proposed and evaluated considering measures such as Feasibility, Performance Cost, System Cost, Issues Solved, New Limitations, and Patient Experience. It has been seen that for group (i), limiting the exercise area is best, since the other options have more limitations, a higher cost, or do not provide a positive experience for the patient; for group (ii), making the main hand non-interactive is the most beneficial, since it allows the patient to still see their main hand, but it does not play a role in the rehabilitation exercise; and for group (iii), showing a ghost hand in the form of an automatic tutorial, will draw attention to the exercise area and help the patient focus.

Our Future Work will focus on the extension of the tests to consider more patients, more rehabilitation exercises, more pathologies, and other devices. In addition, system usability with respect to the rehabilitators’ experience will be evaluated. We also plan to extend authoring tools to provide more rehabilitation exercises.

## Data Availability

The datasets generated during and/or analysed during the current study are available from the corresponding author on reasonable request.
